# Native Tertiary Structure and Nucleoside Modifications Suppress tRNA’s Intrinsic Ability to Activate the Innate Immune Sensor PKR

**DOI:** 10.1371/journal.pone.0057905

**Published:** 2013-03-04

**Authors:** Subba Rao Nallagatla, Christie N. Jones, Saikat Kumar B. Ghosh, Suresh D. Sharma, Craig E. Cameron, Linda L. Spremulli, Philip C. Bevilacqua

**Affiliations:** 1 Department of Chemistry and Center for RNA Molecular Biology, The Pennsylvania State University, University Park, Pennsylvania, United States of America; 2 Department of Chemistry, University of North Carolina, Chapel Hill, North Carolina, United States of America; 3 Department of Biochemistry and Molecular Biology, The Pennsylvania State University, University Park, Pennsylvania, United States of America; The John Curtin School of Medical Research, Australia

## Abstract

Interferon inducible protein kinase PKR is an essential component of innate immunity. It is activated by long stretches of dsRNA and provides the first line of host defense against pathogens by inhibiting translation initiation in the infected cell. Many cellular and viral transcripts contain nucleoside modifications and/or tertiary structure that could affect PKR activation. We have previously demonstrated that a 5′-end triphosphate–a signature of certain viral and bacterial transcripts–confers the ability of relatively unstructured model RNA transcripts to activate PKR to inhibit translation, and that this activation is abrogated by certain modifications present in cellular RNAs. In order to understand the biological implications of native RNA tertiary structure and nucleoside modifications on PKR activation, we study here the heavily modified cellular tRNAs and the unmodified or the lightly modified mitochondrial tRNAs (mt-tRNA). We find that both a T7 transcript of yeast tRNA^Phe^ and natively extracted total bovine liver mt-tRNA activate PKR *in vitro*, whereas native *E. coli*, bovine liver, yeast, and wheat tRNA^Phe^ do not, nor do a variety of base- or sugar-modified T7 transcripts. These results are further supported by activation of PKR by a natively folded T7 transcript of tRNA^Phe^ *in vivo* supporting the importance of tRNA modification in suppressing PKR activation in cells. We also examine PKR activation by a T7 transcript of the A14G pathogenic mutant of mt-tRNA^Leu^, which is known to dimerize, and find that the misfolded dimeric form activates PKR *in vitro* while the monomeric form does not. Overall, the *in vitro* and *in vivo* findings herein indicate that tRNAs have an intrinsic ability to activate PKR and that nucleoside modifications and native RNA tertiary folding may function, at least in part, to suppress such activation, thus serving to distinguish self and non-self tRNA in innate immunity.

## Introduction

Interferon-inducible PKR is a vital component of innate immunity, which provides the first line of defense against pathogens [1], [2]. PKR consists of two functional domains: an N-terminal double stranded RNA (dsRNA) binding domain (dsRBD) comprised of two dsRNA binding motifs (dsRBMs) and a C-terminal kinase domain. These domains are separated by a flexible 20 amino acid linker [3], [4]. The dsRBM recognizes dsRNA with no sequence specificity via minor groove interactions [5], [6], while the kinase domain helps mediate dimerization of PKR as well as possessing the kinase catalytic activity [7]–[9].

Activation of PKR is well known to be promoted by long stretches of dsRNA (>33 base pairs), which may arise as intermediates during viral replication. RNA species of this length are long enough to accommodate a PKR dimer [10]–[12]. Two models of RNA-mediated activation of PKR have been offered: 1) a model in which PKR has intrinsically disordered regions that become ordered upon RNA binding, and 2) an autoinhibition model in which the latent protein is locked into a closed conformation that is relieved upon binding to dsRNA of sufficient length [3], [13]. Activated PKR then phosphorylates translation initiation factor eIF2α, inhibiting translation initiation [14]. This overall process provides essential antiviral and antiproliferative functions for the infected host cell [15].

In addition to the above functions, PKR has been shown to modulate cell-signaling pathways, which alter numerous cellular responses [16]. For instance, PKR regulation has been linked to several diseases, including Huntington’s, Parkinson’s and Alzheimer’s disease [17]–[21]. Furthermore, recent studies indicate that PKR regulates insulin action and metabolism in response to nutrient signals and endoplasmic reticulum stress [22].

A number of different RNAs beyond long, perfect dsRNA activate PKR [1], [23]. For instance, certain highly structured single-stranded viral and cellular RNAs with bulges, imperfect loops, pseudoknots, and single stranded tails can activate PKR [24]–[26]. In addition, largely single-stranded RNA can activate PKR in a 5′-triphosphate dependent manner, which may help distinguish self and non-self RNAs [27]–[29], and this activation can be abrogated by incorporating nucleoside modifications into the transcripts [30], [31]. Several examples of non-canonical RNA activators of PKR include: HIV TAR and HDV RNA dimers [10], [11], domain II and domain III–IV of the internal ribosome entry site (IRES) of HCV [32], [33], the 3′-untranslated regions (UTRs) of several highly structured cytoskeletal mRNAs [34],[35], the 5′-UTR of IFN-γ mRNA [24], [25], and mutant transcripts of the Huntington’s and myotonic dystrophy protein kinase (*DMPK*) genes [18], [36], [37]. These non-canonical RNA activators provide an activating length of 33 bp by either dimerization of the RNA [10], [11], which roughly doubles the length of a given RNA species, or by non-Watson-Crick interactions in the loops of the RNA [1], [25]. In addition, siRNA containing just 19–21 bp can activate PKR, possibly by forming extended RNA species [1], [38], [39].

Another important biological RNA that has so far not been studied with respect to PKR regulation is transfer RNA (tRNA). The folding of tRNAs conforms to a cloverleaf secondary structure and requires magnesium [40] and post-transcriptional modifications [41]–[43] in order to achieve high tertiary structure stability. Studies have shown that an unmodified tRNA transcript of yeast tRNA^Phe^ can be aminoacylated and has a similar lead cleavage pattern, indicating that the tertiary structure of this unmodified T7 tRNA transcript is similar to that of native tRNA [41], [42]. Likewise, the crystal structure of an unmodified *E. coli* tRNA^Phe^ has an overall fold that is nearly identical to that of the native tRNA [44].

Several studies suggest that tRNAs have a tendency to dimerize and that such dimers are separable by analytical techniques. Examples of dimerizing tRNAs include tRNA^Tyr^ [45] and tRNA^Glu^ [46] from *E.coli*; tRNA^Ala^ from yeast [47]; and a mutant form of mitochondrial tRNA^Leu^ [48], [49]. Given that modifications in tRNA strengthen tertiary structure [41]–[43], one function of modifications might be to favor native RNA tertiary structure and thereby minimize tRNA dimerization. This observation could be related to innate immunity given previous data indicating that RNA dimerization drives PKR activation [10], [11]. In addition, previous studies suggest that unmodified tRNAs are capable of inducing an innate immune response via tumor necrosis factor-alpha (TNF-α) in dendritic cells through Toll-like receptors (TLRs), whereas this induction is abrogated by modified tRNA [50]. The effect of nucleoside modifications and the function of dimers in tRNA have not been probed for regulation of PKR.

We recently reported that naturally occurring nucleoside modifications modulate PKR activation in an RNA structure-specific manner [30]. Introduction of most RNA modifications into a largely single-stranded RNA 47mer, “ssRNA-47” that activates PKR in a 5′-triphosphate-dependent manner [27] were found to abrogate activation, whereas just a few of these modifications abrogated PKR activation by perfectly dsRNA. In the present study, we evaluate PKR activation by incorporating nucleoside modifications into biologically relevant tRNA transcripts, as well as endogenous tRNAs from various organisms. In the cytoplasm of the cell, tRNAs are the most heavily modified RNAs, with approximately 20% of its nucleosides being modified [51]. Since PKR is mostly present in the cytoplasm [52] and based on our previous studies on modified short RNAs and mRNAs [30], [31], we reasoned that modifications in tRNAs may have a significant role in suppressing activation of PKR.

Overall, we find that unmodified tRNAs activate PKR and that this activation is abrogated by incorporating numerous modifications into tRNAs. Moreover, we demonstrate that in some cases, tRNA dimers activate PKR, whereas monomers do not. General implications of RNA lacking nucleoside modifications and native tertiary structure for the innate immune response to pathogens are discussed.

## Results and Discussion

### Most Nucleoside Modifications Abrogate Activation of PKR by tRNA

**Figure 1:**
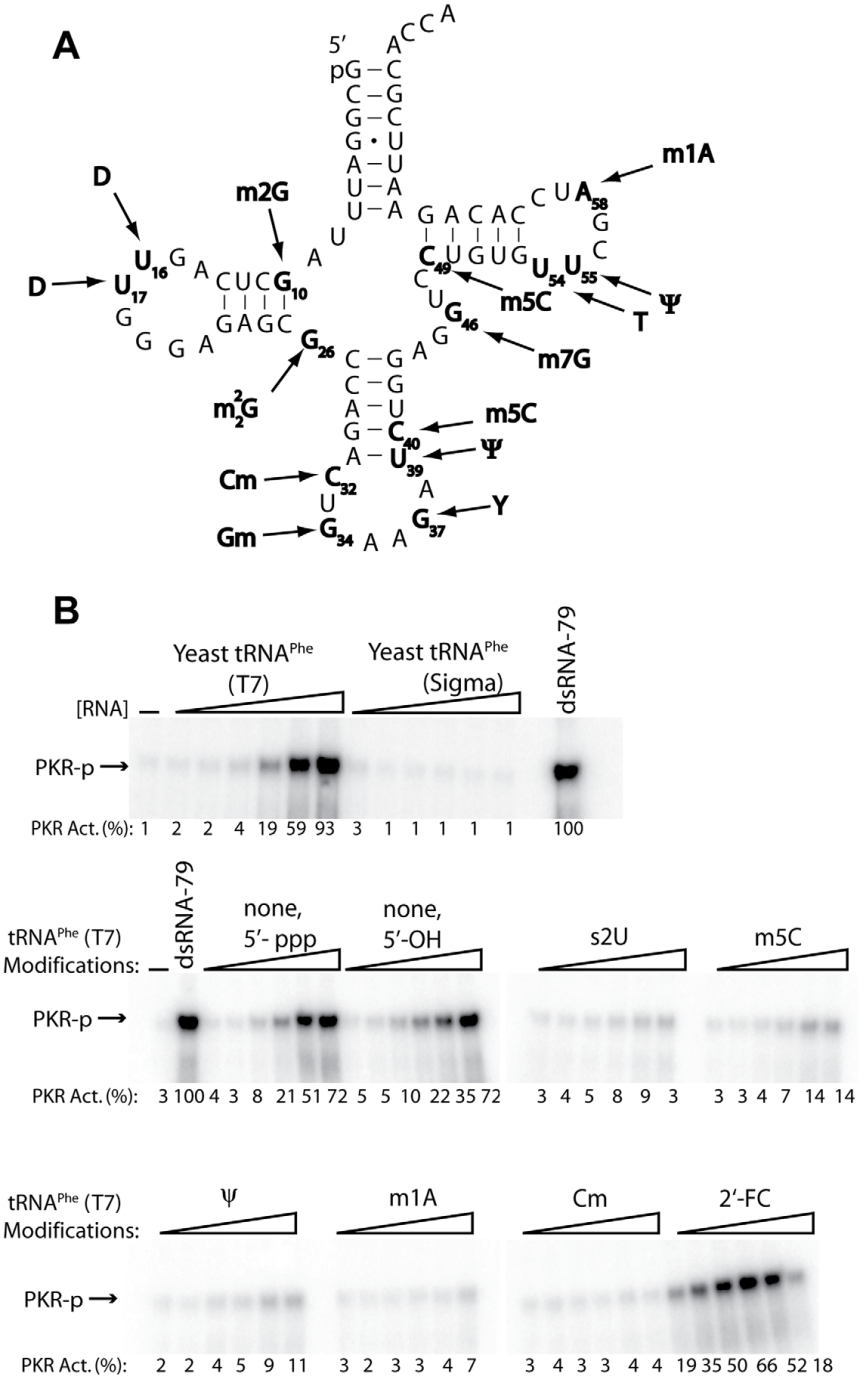
Effect of tRNA modifications on PKR activation. (A) Secondary structure of tRNA^Phe^ in the cloverleaf representation. Positions of modifications are depicted in bold font, where m2G = *N* ^2^-methylguanosine, D = dihydrouridine, m22G = *N* ^2^,*N* ^2^-dimethylguanosine, Cm = 2′-*O*-methylcytidine, Gm = 2′-*O*-methylguanosine, ψ = pseudouridine, m7G = 7-methylguanosine, m5C = 5-methylcytidine, and m1A = 1-methyladenosine, Y = wybutosine. (B) Activation assays for modified tRNAs (10% SDS-PAGE). RNA concentrations are 0.31, 0.63, 1.25, 2.5, 5, and 10 µM. A no RNA control and a positive control of 0.1 µM dsRNA-79 are included. Positions of modifications are depicted, where 5′-ppp = triphosphate at 5′-terminus (present on all transcripts, unless otherwise noted), 5′-OH = hydroxyl at 5′-terminus, s2U = 2-thiouridine, m5C = 5-methylcytidine, 2′-FC = 2′-fluorocytidine, and, m1A, and Cm are as defined in panel A. Phosphorylation activities are provided below each gel lane. Phosphorylation activities were normalized to dsRNA-79 and rounded to the nearest integer.

In order to assess the effect of nucleoside modifications on PKR activation by tRNA, we chose to examine the commercially available phenylalanine-specific tRNA from yeast (tRNA^Phe^) as an RNA substrate (Fig. 1A). At first we compared activation (i.e. phosphorylation) of PKR by the T7 transcript of tRNA^Phe^ and the natively isolated tRNA^Phe^ (Fig. 1B, row 1, compare the two lane sets). (In an innate immune response, PKR autophosphorylates, which allows it to phosphorylate eIF2α which inhibits translation initiation.) The T7 transcript contains a 5′-triphosphate and unmodified bases, whereas the native tRNA has a 5′-monophosphate and modified bases. The T7 transcript activated PKR to almost the same levels as the perfectly double stranded, dsRNA-79, whereas native tRNA^Phe^ did not activate PKR above background at any of the RNA concentrations tested (Fig. 1B). The abrogation of PKR activation by native tRNA^Phe^ may be due to the internal modifications or to lack of a 5′-triphosphate, given that some RNAs require a 5′-triphosphate to activate PKR [27]. To test the role of the 5′-triphosphate, we tested PKR activation by calf intestinal phosphatase (CIP)-treated T7-tRNA^Phe^. Activation of PKR was completely retained by CIP-treated T7-tRNA^Phe^ (Fig. 1B, row 2, compare ‘5′-ppp’ and ‘5′-OH’ lanes), indicating that the 5′-triphosphate is not a factor for PKR activation by T7-tRNA^Phe^. The unimportance of a 5′-triphosphate for tRNA-PKR function is similar to that found for dsRNA and PKR [27]. Overall, these findings suggest that nucleoside modifications may play a key role in abrogating PKR activation by native tRNA^Phe^.

In order to better understand the role of nucleoside modifications in native tRNA-PKR interactions, we tested PKR activation by a series of modified T7-tRNA^Phe^ transcripts. We fully substituted uridines in tRNA^Phe^ with either 2-thiouridine (s2U) or pseudouridine (ψ); cytidine with either 5-methylcytidine (m5C), 2′-*O*-methylcytidine (Cm), or 2′-fluorocytidine (2′-FC); and adenosine with N1-methyladenosine (m1A) (Fig. 1B). These modified nucleosides are commercially available as triphosphates, which are amenable for use in *in vitro* transcription. Transfer RNAs containing the majority of these modifications (s2U, ψ, m5C, Cm, and m1A) revealed significant reduction in PKR activation, the exception being 2′-FC, which showed an *enhancement* in PKR activation. These results indicate that modifications generally abrogate activation of PKR by tRNA, just as observed previously in model RNAs [30]. Moreover, retention of PKR activation by tRNAs containing 2′-FC, which can accept a hydrogen bond, supports the importance of minor groove hydrogen bonding between the dsRBD and tRNA, as previously reported in unmodified dsRNAs [5], [6]. In considering these data, it is important to keep in mind that full substitution of a natural base with a modified one will affect the folding of the tRNA; as such, changes in PKR activation will be due to a combination of direct effects of the modification and indirect effects of the RNA folding. Overall, these findings suggest that tRNA has the potential to activate PKR and thus lead to an innate immune response.

### Mitochondrial tRNAs Activate PKR, Other Native tRNAs do not

**Figure 2:**
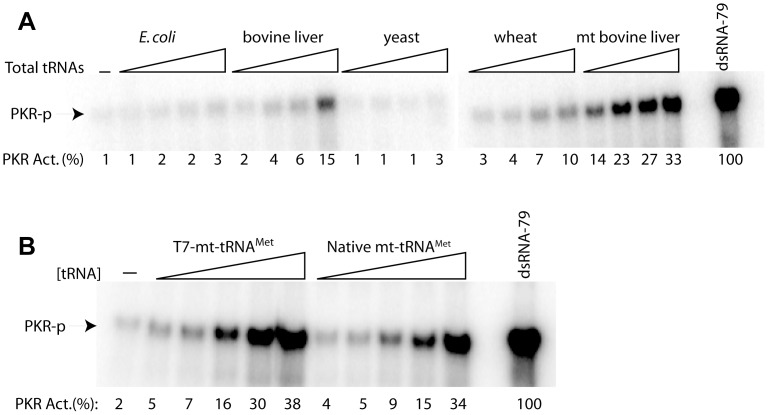
Activation of PKR by tRNAs from various sources. (A) Activation assays for total tRNAs isolated from *E. coli*, bovine liver, yeast, wheat, and mitochondrial bovine liver. RNA concentrations are 0.63, 1.25, 2.5, and 5 µM. (B) PKR activation for T7 transcript and natively isolated mitochondrial tRNAs specific for methionine. RNA concentrations are 0.31, 0.63, 1.25, 2.5, and 5 µM. For each panel, a control of no RNA and a positive control of 0.1 µM dsRNA-79 are included. Phosphorylation activities are provided below each gel lane. Phosphorylation activities were normalized to dsRNA-79 and rounded to the nearest integer.

The level of modification in tRNA varies depending on the origin of the tRNA. For example, cytoplasmic tRNAs are heavily modified (∼14 sites/tRNA), whereas mitochondrial tRNAs generally contain relatively few modifications (∼3 sites/tRNA) and have less variety in the modifications observed [53]. In particular, tRNAs from *E. coli*, bovine liver, yeast, and wheat contain many modifications as compared to mitochondrial bovine liver tRNAs [54]. To further test our observation that nucleoside modifications in tRNA influence PKR activation, we tested PKR activation by total tRNAs from different organisms, including *E. coli*, bovine liver, yeast, wheat and bovine liver mitochondria. All of these tRNAs are available commercially except for mitochondrial tRNA, which was isolated using published procedures [55]. As shown in Figure 2A, total mitochondrial bovine liver tRNA significantly activated PKR, while total tRNAs from other organisms did not. For example, at a 1.25 µM concentration, tRNAs from *E. coli*, bovine liver, yeast and wheat showed lower activation of PKR by ∼22-, 7-, >25- and 7-fold, respectively, as compared to mitochondrial tRNA. These observations strongly suggest that nucleoside modifications in these tRNAs play a major role in the abrogation of PKR activation, either directly or through their effects on tRNA structure.

We next compared PKR activation by a natively isolated methionine-specific mitochondrial tRNA (mt-tRNA^Met^) to activation by an *in vitro* transcribed version (T7-mt-tRNA^Met^). Remarkably, both of these tRNAs potently activated PKR, having activation profiles similar to each other and to the total mt bovine liver preparation, although there was somewhat less activation by the native mt-tRNA, perhaps reflecting a slight suppression of activation by its few modifications (Fig. 2B). Because *in vitro* transcribed T7-mt-tRNA^Met^ has no modifications and native mt-tRNA^Met^ has very few, we believe that the ability of these tRNAs to activate PKR resides in the little or no modification present in them. This interpretation supports the notion that modifications in cytoplasmic tRNA play a significant role in minimizing PKR activation either directly or through effects on tRNA structure. Lastly, these results further support our earlier conclusion that PKR activation by tRNA is not 5′-triphosphate-dependent in that neither of these tRNAs has a 5′-triphosphate yet they are both potent activators: the unmodified T7-mt-tRNA^Met^ has a hammerhead ribozyme-generated 5′-hydroxyl while the natively isolated mt-tRNA^Met^ has a 5′-monophosphate.

### Activation of PKR by a mt-tRNA Dimer

Previous reports indicated that tRNAs are intrinsically capable of forming dimers [45]–[49]. This tendency may be due to their simple secondary structure, which is always at least partially self-complementary. We, therefore, tested our various tRNAs for dimer formation. When we performed native gel analysis on unmodified radiolabeled T7 yeast tRNA^Phe^ we detected a few percent dimer, which, while more than detected in naturally modified yeast tRNA^phe^, was not enough to purify (Fig. S1 in File S1). We then turned to a human mitochondrial tRNA.

**Figure 3:**
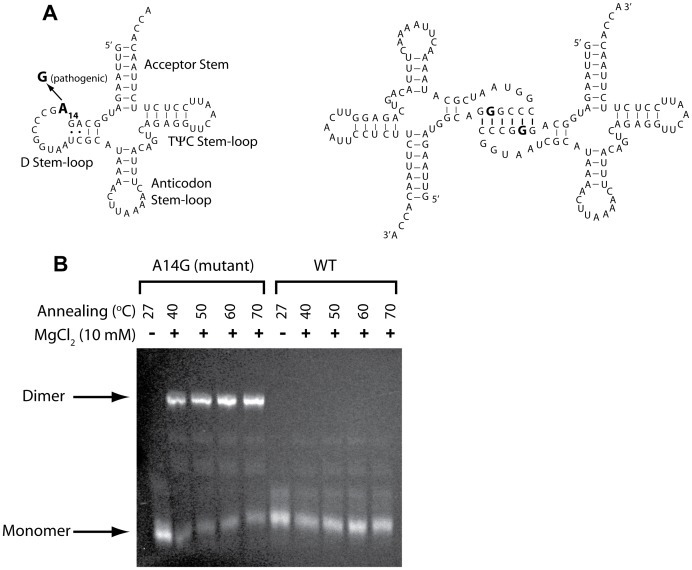
Analysis of hs mt-tRNA^Leu^ A14G mutant dimer formation. (A) Secondary structure of hs mt-tRNA^Leu^ in the cloverleaf representation with the four stem-(loops) named (left-hand panel). In wild-type, the 14^th^ position (bold font) is ‘A’, and in the pathogenic tRNA it is ‘G’. Schematic representation of the previously reported dimeric complex formed by the pathogenic A14G hs mt-tRNA^Leu(UUR)^ mutant (right-hand panel), adopted from ref. [49]. (B) Dimerization assay for WT and A14G pathogenic hs mt-tRNA^Leu^. 2 µM tRNA samples in 1XTE were annealed for 5 min at the indicated renaturation temperature, MgCl_2_ was added to 10 mM, and then cooled on ice. Samples were subjected to native gel electrophoresis with running buffer 0.5X TB (45 mM Tris base and 45 mM boric acid) and stained with ethidium bromide. Positions of the previously characterized [48] dimeric species (present only for the mutant) and the monomeric species are indicated.

Human mitochondrial tRNA^Leu^ has been reported to form a stable dimer when the pathogenic A14G mutation occurs [48]. This dimer can be separated from the monomer using native PAGE purification, and its secondary structure has been previously probed and shown to involve six GC base pairs in the D stem-loop (Fig. 3A) [49]. We first confirmed dimerization of mt-tRNA^Leu^ (A14G) using native gel electrophoresis under slightly modified conditions. As shown in Figure 3B, the dimer band is only observed in the A14G lanes, with more than 50% of the tRNA forming a dimer under all temperature renaturation conditions. (The identity of this species as a dimer has been confirmed previously by native gel markers [48].).

**Figure 4:**
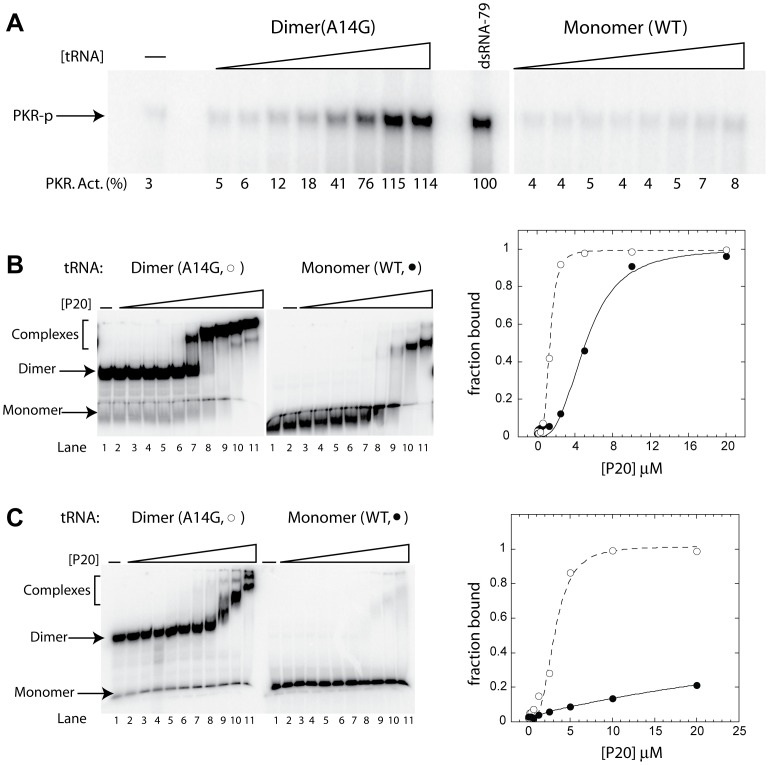
PKR is activated by and binds tightly to A14G mt-tRNA^Leu^ dimers. (A) Activation assays for native gel purified WT monomer and A14G dimer of mt-tRNA^Leu^. RNA concentrations are 0.04, 0.08, 0.16, 0.32, 0.63, 1.25, 2.5, and 5 µM. A no RNA control and a positive control of 0.1 µM dsRNA-79 are included. Phosphorylation activities are provided below each gel lane. Phosphorylation activities were normalized to dsRNA-79 and rounded to the nearest integer. (B, C) Binding assays for dsRBD of PKR (P20) and WT and A14G mt-tRNA^Leu^. Trace amount of 5′-^32^P-labeled tRNA was used in the binding experiments. Protein concentrations are 0, 0.04, 0.08, 0.16, 0.32, 0.63, 1.25, 2.5, 5, 10 and 20 µM and are present in Lanes 1 to 11, respectively. For one set of binding experiments (B) herring sperm DNA competitor was used (0.1 mg/mL) and for the other (C) tRNA competitor was used (0.1 mg/mL). Binding constants for A14G (○) and WT (•) in DNA competitor were 1.3 and 5.1 µM with Hill coefficients of 3.7 and 2.9 respectively, and in the presence of tRNA competitor they were 3.1 and >20 µM with Hill coefficients of 3.1 and undetermined, respectively. Higher Hill coefficients correlate with multiple bands of lower mobility on native gels, especially in Panel C (14G); this may relate to the dimer having enough binding registers to accommodate multiple copies of P20 at one time.

Next, we investigated activation of PKR by dimers and monomers of mitochondrial tRNA^Leu^. The dimer was annealed and purified as described in the Materials and Methods. Purified dimer was stable when stored at −20°C, as checked by ^32^P-radiolabeling analysis (e.g. Fig. 4B, 4C). In addition, we isolated the mt-tRNA^Leu^ (WT) monomer on a native gel in order to eliminate any dimeric form.

Upon testing the activation of PKR by dimers and monomers of mt-tRNA^Leu^, the tRNA dimer activated PKR, whereas the tRNA monomer did not (Fig. 4A). In fact, no appreciable activation of PKR by tRNA monomer was observed even at concentrations up to 5 µM. In addition, the dimer tRNA (A14G) activated PKR to the same levels as perfect dsRNA-79 (Fig. 4A). This result clearly shows that the dimeric form of this tRNA is an activator of PKR.

It has been shown that 16 bp of dsRNA is required for binding to PKR and that 30 bp is required for activation [5], [12], [56], [57]. Moreover, the dsRBD can tolerate non-Watson-Crick base pairs, and noncontiguous helical stems can help PKR dimerize for activation [1], [58]. In the dimeric form of mt-tRNA^Leu^, 6 extra base pairs form (Fig. 3A) [49]. Thus, while only ∼20 base pairs are present in monomeric mt-tRNA^Leu^, ∼40 base pairs are present in dimeric mt-tRNA^Leu^, which may be sufficient for PKR binding and activation.

Next, we analyzed binding of PKR to both the monomeric and dimeric forms of mt-tRNA^Leu^. Native mobility-shift experiments were carried out as previously described, using P20, the dsRBD of PKR [5]. Appreciable binding was observed for dimer (*K*_d_∼1 µM) compared to monomer (*K*_d_∼5 µM) in the presence of 0.1 mg/mL herring sperm DNA (Fig. 4B). Moreover, the binding ability of the monomer was completely abolished (*K*_d_ >20 µM) when 0.1 mg/mL native yeast tRNA^Phe^ was used as a competitor, while the binding ability of the dimer was reduced just 2.4-fold (*K*_d_∼3 µM) (Fig. 4C). These results strongly suggest that the dimer is capable of binding PKR with high affinity as compared to the monomer. Overall, the approximate doubling of the number of base pairs present in the tRNA dimer helps promote tight binding and activation of PKR, as observed for other RNAs [10], [11].

### Activation of PKR by tRNA *in vivo*

**Figure 5:**
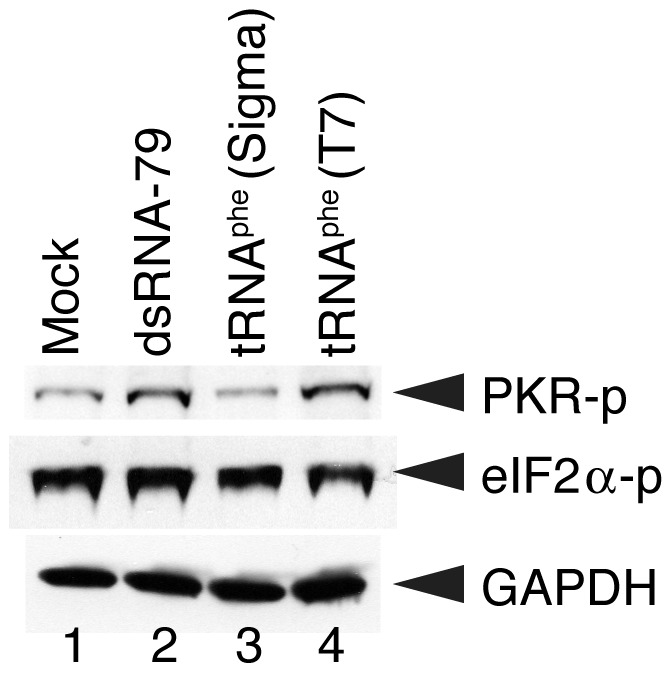
Activation of PKR by yeast tRNA *in vivo*. Cells were plated 8 h before transfection. Cells were transfected for 16 h with 2 µg of either 79 bp dsRNA (lane 2), native tRNA^Phe^ (Sigma) (lane 3), or unmodified yeast tRNA^Phe^ (T7) (lane 4), except in the ‘mock’ lane (lane 1) which had no RNA. The tRNA and dsRNA-79 were prepared as per *in vitro* activation experiments. The ‘mock’ and ‘dsRNA-79′ lanes serve as negative and positive controls, respectively, for PKR phosphorylation. Proteins were denatured in SDS buffer and resolved in 10% SDS-PAGE. Phosphorylated PKR (PKR-p), phosphorylated eIF2α (eIF2α-p), and GAPDH (loading control) were identified by Western blotting.

We wanted to test whether PKR is activated by unmodified tRNA in cells. The Huh-7 cell line was chosen because it can produce interferon (IFN)-α/β and has the capacity to signal from the IFN-α/β receptor [27]. These cells can thus lead to PKR phosphorylation without the addition of IFN. Transfection with the positive control of dsRNA-79 induced activation of PKR (Fig. 5, lane 2), while the mock transfection with RNA omitted did not (Fig. 5, lane 1). Transfections of Huh-7 cells with a T7 transcript of yeast tRNA^phe^ or natively isolated yeast tRNA^phe^ were also conducted. The T7 transcript induced activation of PKR to the same level as the same amount of dsRNA-79 (Fig. 5, compare lanes 4 and 2, respectively), while native tRNA^phe^ did not activate PKR above the background found in the mock lane (Fig. 5, compare lanes 3 and 1, respectively). These results parallel those found *in vitro* (Fig. 1B) and indicate that tRNAs have an intrinsic ability to activate PKR both *in vitro* and *in vivo*. It is important to note that unmodified and native yeast tRNA^phe^ are thought to have the same tertiary structure, [41], [42], [44] suggesting that tRNA modifications may play a direct role in suppressing PKR activation in cells, at least in this case.

Lastly, we note that eIF2α was phosphorylated to a similar level in all four of the conditions tested: mock, dsRNA-79, tRNA^phe^ (Sigma), and tRNA^phe^ (T7) (Fig. 5). Similar eIF2α behavior has been noticed in several prior studies [27], [59]–[61] and may reflect the active signaling to the IFN-α/β receptor present in Huh-7 cells. Nonetheless, it is clear that only unmodified tRNA^phe^ induces activation of PKR, which, in turn, should lead to phosphorylation of eIF2α.

### Conclusion

PKR was originally described as the double-stranded RNA-activated protein kinase [62]. While this moniker still holds, it has become clear that PKR is activated by much more than dsRNA. RNAs with complex secondary and tertiary structures activate PKR [1], [58]. Moreover, dimerization of RNA drives PKR activation, primarily because it approximately doubles the number of base pairs that PKR can interact with and because RNA dimers tend to have extensive helical regions and less tertiary structure [10], [11]. In addition, most nucleoside modifications abrogate PKR activation [30], [31].

It then becomes of interest to ask not only which RNAs activate PKR, but which RNAs *do not* and why? For instance, have cellular RNAs been selected so that they do not spuriously activate PKR? We showed in this study that unmodified or lightly modified tRNAs, such as mt-tRNA, have an intrinsic ability to activate PKR *in vitro*, but that tRNAs that are heavily modified suppress this ability. Moreover, the dimer form of an unmodified tRNA^Leu^ transcript can activate PKR *in vitro*. Dimerization of tRNA has been long known [45]–[49], however functional significance of such dimers has remained obscure. Given that nucleoside modifications strengthen native tRNA tertiary structure [41]–[43], which would resist dimerization, it is possible that RNA modifications serve, at least in part, to help distinguish self from non-self tRNAs by resisting RNA misfolding.

We do note that it is unclear at present whether native PKR-activating mt-tRNAs–such as mitochondrial bovine liver total tRNAs and mt-tRNA^Met^–activate PKR as dimers or monomers. Observation that the T7 transcript of tRNA^Leu^ monomer did not activate PKR *in vitro* might seem to indicate that tRNA dimerization is critical for PKR activation; however it has been shown elsewhere that this non-activating transcript does not fold unless its synthetase is present [63]. Moreover, we were unable to detect any dimers of the PKR-activating mt-tRNA^Met^ or other mt-tRNAs *in vitro* (Fig. S2 in File S1). On a related note, an unmodified T7 human mt-tRNA^Met^ transcript, which is very close in sequence to the PKR-activating bovine version, adopts a cloverleaf structure [64]. This observation strengthens the importance of having native secondary structure in monomeric tRNA in order to activate PKR. This then suggests that RNA modifications play *dual* roles in suppressing activation of PKR by tRNA: 1.) They may help to abrogate activation when tRNA is a monomer with native secondary structure, which is supported by *in vivo* activation by natively folded yeast T7 tRNA^Phe^, and 2.) They may stabilize native tertiary structure to prevent the tRNA from dimerizing, which potentially activate PKR. Indeed, a role for RNA modifications in suppressing PKR activation has been reported previously for a non-dsRNA both *in vitro* and *in vivo* [30], [31].

That tRNAs capable of activating PKR exist in the human cell but reside where PKR does not (*i.e.* in the mitochondria) is consistent with the absence of selective pressure on such tRNAs to avoid activating the innate immune system, although other functions of the modifications such as influencing native folding and the binding of proteins are almost certainly important. This possibility supports the notion that modification in cellular tRNAs function, at least in part, to suppress intrinsic activation of innate immune responses. It will be of interest to test whether there is a connection between tRNA from pathogens and PKR activation, as related to the innate immune system. In addition, it will be of interest to test whether other classes of cellular RNAs have an intrinsic ability to activate PKR or other innate immune sensors, and if certain cellular factors–for example, RNA modifications, tertiary structure, or protein binding function, at least in part, to suppress an innate immune response. The existence of the ability to activate the innate immune system intrinsic to the sequence and structure of a cellular RNA suggests the potential for rich regulation of innate immunity in response to stress, disease, or other exogenous signals that could function to unmask these suppressed RNA signatures.

## Materials and Methods

### Protein Expression and Purification

Full length PKR containing an N-terminal (His)_6_, which does not interfere with binding or activation [5], [57], was cloned into pET-28a (Novagen, Inc.) and transformed into *E. coli* BL21 (DE3) Rosetta cells (Novagen, Inc.) as described [5], [27], [57]. Briefly, cells were sonicated and the protein was purified by a Ni^2+^-agarose column (Qiagen, Inc.). PKR was dialyzed into storage buffer of 10 mM Tris-HCl (pH 7.6), 50 mM KCl, 2 mM Mg(OAc)_2_, 10% glycerol, and 7 mM β-mercaptoethanol (βME). Prior to PKR activation assays, isolated protein was treated with λ-protein phosphatase (λ-PPase) (NEB) as described below.

### RNA Preparation and Purification

Unmodified and s2U-, m5C-, ψ-, m1A-, and Cm-modified tRNAs were prepared for *in vitro* and *in vivo* experiments and purified as reported [30]. Briefly, a T7 transcription was conducted (Ambion) in which the corresponding modified nucleoside triphosphate was completely substituted in the transcription reaction. The RNAs were purified by denaturing polyacrylamide gel electrophoresis (PAGE), identified by UV shadowing, excised, eluted overnight, ethanol precipitated, dissolved in TE [10 mM Tris-HCl (pH 7.5), 1 mM EDTA], and frozen at −20°C. RNA concentrations were determined spectrophotometrically. 2′-FU-containing tRNA was prepared by an Epicentre T7 transcription (Durascribe) kit, as previously reported [30], and the transcript was purified as described above.

*E. coli*, bovine liver, yeast and wheat total and tRNA^Phe^-specific tRNAs were purchased from Sigma. Total bovine liver mitochondrial tRNAs and methionine-specific mitochondrial tRNA (mt-tRNA^Met^) were isolated as previously described using an oligonucleotide with the sequence 5′ TAGTACGGGAAGGATATAAACCAACATTTTCGGG-biotin [65].

Sequences of T7-transcripts of tRNAs are as follows:

T7-tRNA^Phe^ : GCGGAUUUAGCUCAGUUGGGAGAGCGCCAGACUGAAGAUCUGGA

GGUCCUGUGUUCGAUCCACAGAAUUCGCACCA

T7-Mt-tRNA^Leu^(WT): UUAAGAUGGCAGAGCCCGGUAAUCGCAUAAAACUUAAAACU

UUACAGUCAGAGGUUCAAUUCCUCUUCUUAACACCA

T7-mt-tRNA^Leu^(A14G): GUUAAGAUGGCAGGGCCCGGUAAUCGCAUAAAACUUAAAACU

UUACAGUCAGAGGUUCAAUUCCUCUUCUUAACACCA

T7-mt-tRNA^Met^ (bovine) AGUAAGGUCAGCUAAUUAAGCUAUCGGGCCCAUACCCCGAAAAUGUU

GGUUUAUAUCCUUCCCGUACUACCA

(Note that this transcript was prepared from a hammerhead construct to allow efficient transcription of the gene carrying a 5′A residue, and so has a 5′-OH start) [66].

T7 transcripts were labeled at their 5′-ends as needed by first treating with calf intestinal phosphatase (CIP), followed by polynucleotide kinase (PNK) in the presence of [γ-^32^P]ATP. Native tRNAs from Sigma and native mt-tRNA^Met^ were labeled at their 3′-ends using T4 RNA ligase (NEB) and [^32^P]-pCp.

### tRNA Dimerization Assay

Native gels contained 10% of 29∶1 (acrylamide: bis) crosslinking polyacrylamide and were used to analyze the mobility differences between monomer and dimers of tRNAs. The buffer in both the gel and running electrophoresis was 0.5×TB [50 mM Tris base, 40 mM boric acid]. Samples were fractionated between 4 and 12 h at 300 V and 16°C. Briefly, 2 µM tRNA samples in TE were renaturated at 40, 50, 60 or 70°C for 5 min; MgCl_2_ was immediately added to a final concentration of 10 mM; and RNA was cooled on ice. Next, samples were subjected to native gel electrophoresis with 0.5×TB running buffer and stained with ethidium bromide for visualization. (We note that employing 0.5×TBE showed reduced formation of the dimer on native gels, which may be due to sequestration of magnesium ions by EDTA during the native gel run.).

Preparative native gel electrophoresis of tRNA dimers for PKR activation assays followed a similar protocol. The dimer was prepared by annealing 10 µM mt-tRNA^Leu^ (A14G) at 70°C followed by fractionation on a 0.5×TB native gel and a crush and soak procedure similar to previously described [10].

### Native Gel Mobility Shift Assay

Binding of dsRBD (P20) to tRNA monomer and dimer was carried out by native gel mobility shift assays as described [5]. Briefly, a trace amount of 5′-end labeled RNA was incubated with various concentrations of P20 at room temperature for 10 min in binding buffer [BB: 25 mM Hepes-KOH (pH 7.5), 10 mM NaCl, 5% glycerol, 5 mM dithiothreitol, 0.1 mM EDTA and 0.1 mg/mL tRNA^Phe^ (Sigma) or 0.1 mg/mL herring sperm DNA(Promega)]. Binding reactions were loaded onto a running 10% (29∶1 acrylamide/bis) native gel. The gel and the running buffer contained 0.5×TB, and electrophoresis was performed at 300 V at 16°C for 2 h. Gels were exposed to a storage screen and analyzed on a PhosphorImager (Molecular Dynamics).

### PKR Activation Assays *in vitro*

Modified and unmodified tRNAs, total tRNAs, and monomer and dimers of tRNAs were tested for their ability to activate PKR autophosphorylation. Prior to use in activation assays, T7 and Sigma tRNA^Phe^ were heated at 90°C for 3 min and cooled at room temperature. All other tRNAs were used without any renaturation prior to an activation assay, including native gel-isolated monomer and dimer of pathogenic tRNAs, total tRNAs, and native and T7 transcripts of mt-tRNA^Met^. Activation assays were carried out largely as previously described [27], [57]. Briefly, purified PKR was dephosphorylated with λ-PPase (NEB), quenched by the addition of freshly prepared sodium orthovanadate, and incubated with [γ-^32^ P] ATP for 10 min at 30°C. The time of 10 min was chosen because this is in the plateau region of phosphorylation versus time plots [27]. Reactions were quenched by SDS loading buffer. Samples were heated at 95°C for 5 min and loaded onto a 10% SDS PAGE gel (Pierce). Gels were exposed to a storage PhosphorImager screen, and bands were quantified on a PhosphorImager (Molecular Dynamics). In all experiments, data were normalized to the counts in a 0.1 µM of dsRNA-79 lane. Note that all activation assays in the same figure were conducted on the same day and exposed to the same PhosphorImager screen for the same length of time and so can be referenced to the same dsRNA-79 reference lane.

### PKR Activation Assays *in vivo*

Huh-7 cells were maintained as described [27]. The transfection procedure was similar to previously described, [27] with the following exceptions. Lysates were prepared from 1×10^6^ Huh-7 cells that were transfected for 16 h with 2 µg of the respective RNA, prepared and renatured as described above, using DOTAP (N-[1-(2, 3-dioleoyloxy)propyl]-N, N, N-trimethylammonium methylsulfate) as the transfection reagent (Sigma), which has been used recently for similar transfections [67]. The RNA was omitted from the ‘mock’ transfection control. Cells were lysed using lysis buffer containing 50 mM Tris-HCl pH 7.4, 100 mM NaCl, 0.5% sodium deoxycholate, 1% NP-40, 0.1% SDS and 1/100 Protease inhibitor cocktail (Calbiochem). The lysates were further treated with RQ1 DNase (Promega) at 37°C for 5 min and clarified by centrifugation at 16,000 g for 10 min. A total of 25 µL of samples were loaded for PKR-p (PKR phosphorylated at T446) and eIF2α-p (eIF2α phosphorylated at S51) immunostaining, while 5 µL of sample was loaded for glyceraldehyde-3-phosphate dehydrogensase (GAPDH) immunostaining. Western blotting was performed on samples using rabbit monoclonal antibody against PKR-pT446 (Epitomics) and eIF2α-pS51 (Epitomics) at 1∶1000 dilution, and mouse monoclonal antibody GAPDH (Fitzgerald) at 1∶10,000 dilution for loading control.

## Supporting Information

File S1 **Supplemental material.** Figure S1. Dimerization assay of unmodified yeast tRNA^Phe^ (T7) and native yeast tRNA^Phe^ (Sigma). tRNA samples (2 µM) plus trace radiolabeled tRNA in 1X TE were annealed for 3 min at 90°C, MgCl_2_ was added to 5 mM, and then cooled to room temperature. Samples were subjected to native gel electrophoresis with running buffer 1× THEN_100_M_5_ (33 mM Tris (base form), 66 mM Hepes (acid form) (pH 7.5), 0.1 mM EDTA, 100 mM NaCl, and 5 mM MgCl_2_). The percent dimerization is indicated in each lane below the gel. Very little dimer forms for either tRNA, although there is somewhat more dimer in the T7 transcript. Figure S2. Dimerization assay of unmodified mt-tRNA^Met^ (T7) and native mt-tRNA^Met^. Either 0.625 µM (Lanes 1, 3, 5) or 5 µM (Lanes 2, 4, 6–8) tRNA samples plus trace radiolabeled RNA in 1X TE were renatured in lanes 1–6, as follows: samples were annealed for 5 min at 70°C, MgCl_2_ was added to 10 mM, and then cooled on ice. Lanes 7 and 8 were not renatured. Samples were subjected to native gel electrophoresis with a running buffer of 0.5× TB. tRNAs were loaded as follows: Lanes 1, 2, and 7, native mt-RNA^Met^; Lanes 3, 4, and 8, unmodified mt-tRNA^Met^; Lanes 5 and 6, native yeast tRNA^Phe^ (as per Fig. S1 in File S1). No significant dimer formed for any of these tRNAs under any of the conditions tested. The small amount of a slower migrating band in lanes 3, 4, and 8 is likely a minor amount of tRNA with uncleaved hammerhead.
(PDF)
