# Supplementary material for: Native Tertiary Structure and Nucleoside Modifications Suppress tRNA’s Intrinsic Ability to Activate the Innate Immune Sensor PKR
Source: PLoS One. 2013 Mar 4;8(3):e57905. doi: 10.1371/journal.pone.0057905 (PMC3587421; doi:10.1371/journal.pone.0057905)
Supplement: File S1 — Supplemental material. Figure S1. Dimerization assay of unmodified yeast tRNAPhe (T7) and native yeast tRNAPhe (Sigma). tRNA samples (2 µM) plus trace radiolabeled tRNA in 1X TE were annealed for 3 min at 90°C, MgCl2 was added to 5 mM, and then cooled to room temperature. Samples were subjected to native gel electrophoresis with running buffer 1× THEN100M5 (33 mM Tris (base form), 66 mM Hepes (acid form) (pH 7.5), 0.1 mM EDTA, 100 mM NaCl, and 5 mM MgCl2). The percent dimerization is indicated in each lane below the gel. Very little dimer forms for either tRNA, although there is somewhat more dimer in the T7 transcript. Figure S2. Dimerization assay of unmodified mt-tRNAMet (T7) and native mt-tRNAMet. Either 0.625 µM (Lanes 1, 3, 5) or 5 µM (Lanes 2, 4, 6–8) tRNA samples plus trace radiolabeled RNA in 1X TE were renatured in lanes 1–6, as follows: samples were annealed for 5 min at 70°C, MgCl2 was added to 10 mM, and then cooled on ice. Lanes 7 and 8 were not renatured. Samples were subjected to native gel electrophoresis with a running buffer of 0.5× TB. tRNAs were loaded as follows: Lanes 1, 2, and 7, native mt-RNAMet; Lanes 3, 4, and 8, unmodified mt-tRNAMet; Lanes 5 and 6, native yeast tRNAPhe (as per Fig. S1 in File S1). No significant dimer formed for any of these tRNAs under any of the conditions tested. The small amount of a slower migrating band in lanes 3, 4, and 8 is likely a minor amount of tRNA with uncleaved hammerhead. (PDF) [file pone.0057905.s001.pdf]

Supplemental Material to:

Native tertiary structure and nucleoside modifications suppress tRNA's intrinsic ability to activate the innate immune sensor PKR

Subba Rao Nallagatla[1,4], Christie N. Jones[2,5], Saikat Kumar B. Ghosh[1], Suresh D. Sharma[3], Craig E. Cameron[3], Linda L. Spremulli[2] and Philip C. Bevilacqua[1,*]

[1]Department of Chemistry and Center for RNA Molecular Biology, The Pennsylvania State University, University Park, Pennsylvania, United States of America.

[2]Department of Chemistry, University of North Carolina, Chapel Hill, North Carolina, United States of America.

[3]Department of Biochemistry and Molecular Biology, The Pennsylvania State University, University Park, Pennsylvania, United States of America.

[4]Present address: AuraSense Therapeutics, Skokie, Illinois, United States of America.

[5]Present address: Department of Chemistry, Trinity University, San Antonio, Texas, United States of America.

*Author to whom correspondence should be addressed: Phone: (814) 863-3812; Fax: (814) 865-2927; e-mail: pcb5@psu.edu

Table of Contents:

Figure S1. Dimerization assay of unmodified yeast $tRNA^{Phe}$ (T7) and native yeast $tRNA^{phe}$.

Figure S2. Dimerization assay of unmodified mt-$tRNA^{Met}$ (T7) and native mt-$tRNA^{Met}$.

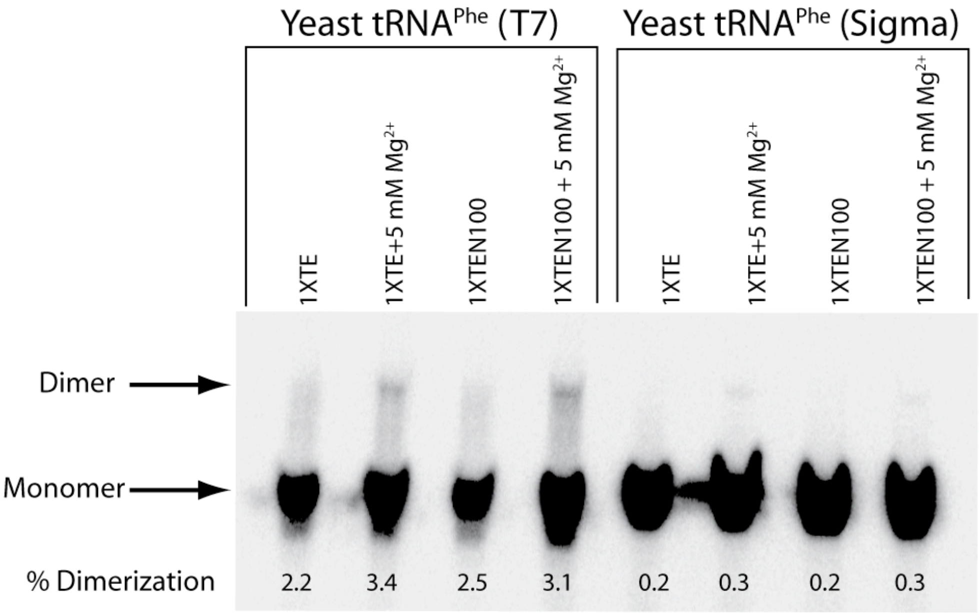

Figure S1. Dimerization assay of unmodified yeast $tRNA^{Phe}$ (T7) and native yeast $tRNA^{Phe}$ (Sigma). tRNA samples (2 μM) plus trace radiolabeled tRNA in 1X TE were annealed for 3 min at 90 °C, $MgCl_2$ was added to 5 mM, and then cooled to room temperature. Samples were subjected to native gel electrophoresis with running buffer 1x $THEN_{100}M_5$ (33 mM Tris (base form), 66 mM Hepes (acid form) (pH 7.5), 0.1 mM EDTA, 100 mM NaCl, and 5 mM $MgCl_2$). The percent dimerization is indicated in each lane below the gel. Very little dimer forms for either tRNA, although there is somewhat more dimer in the T7 transcript.

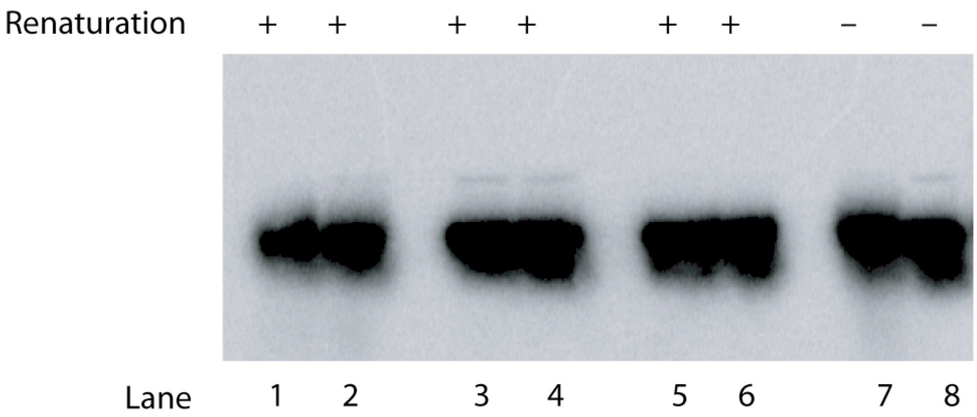

Figure S2. Dimerization assay of unmodified mt-tRNA^{Met} (T7) and native mt-tRNA^{Met}. Either 0.625 μM (Lanes 1, 3, 5) or 5 μM (Lanes 2, 4, 6-8) tRNA samples plus trace radiolabeled RNA in 1X TE were renatured in lanes 1-6, as follows: samples were annealed for 5 min at 70 ^{o}C, $MgCl_2$ was added to 10 mM, and then cooled on ice. Lanes 7 and 8 were not renatured. Samples were subjected to native gel electrophoresis with a running buffer of 0.5x TB. tRNAs were loaded as follows: Lanes 1, 2, and 7, native mt-RNA^{Met}; Lanes 3, 4, and 8, unmodified mt-tRNA^{Met}; Lanes 5 and 6, native yeast tRNA^{Phe} (as per Fig. S1 in File S1). No significant dimer formed for any of these tRNAs under any of the conditions tested. The small amount of a slower migrating band in lanes 3, 4, and 8 is likely a minor amount of tRNA with uncleaved hammerhead.
